# Post-operative glioblastoma multiforme segmentation with uncertainty estimation

**DOI:** 10.3389/fnhum.2022.932441

**Published:** 2022-11-03

**Authors:** Michal Holtzman Gazit, Rachel Faran, Kirill Stepovoy, Oren Peles, Reuben Ruby Shamir

**Affiliations:** Novocure, Haifa, Israel

**Keywords:** Tumor Treating Fields, glioblastoma multiform, MRI, segmentation, treatment planning

## Abstract

Segmentation of post-operative glioblastoma multiforme (GBM) is essential for the planning of Tumor Treating Fields (TTFields) treatment and other clinical applications. Recent methods developed for pre-operative GBM segmentation perform poorly on post-operative GBM MRI scans. In this paper we present a method for the segmentation of GBM in post-operative patients. Our method incorporates an ensemble of segmentation networks and the Kullback–Leibler divergence agreement score in the objective function to estimate the prediction label uncertainty and cope with noisy labels and inter-observer variability. Moreover, our method integrates the surgery type and computes non-tumorous tissue delineation to automatically segment the tumor. We trained and validated our method on a dataset of 340 enhanced T1 MRI scans of patients that were treated with TTFields (270 scans for train and 70 scans for test). For validation, we developed a tool that uses the uncertainty map along with the segmentation result. Our tool allows visualization and fast editing of the tissues to improve the results dependent on user preference. Three physicians reviewed and graded our segmentation and editing tool on 12 different MRI scans. The validation set average (SD) Dice scores were 0.81 (0.11), 0.71 (0.24), 0.64 (0.25), and 0.68 (0.19) for whole-tumor, resection, necrotic-core, and enhancing-tissue, respectively. The physicians rated 72% of the segmented GBMs acceptable for treatment planning or better. Another 22% can be edited manually in a reasonable time to achieve a clinically acceptable result. According to these results, the proposed method for GBM segmentation can be integrated into TTFields treatment planning software in order to shorten the planning process. To conclude, we have extended a state-of-the-art pre-operative GBM segmentation method with surgery-type, anatomical information, and uncertainty visualization to facilitate a clinically viable segmentation of post-operative GBM for TTFields treatment planning.

## Introduction

Glioblastoma multiforme (GBM) is the most frequent and lethal malignant brain tumor in adults. Tumor Treating Fields (TTFields) therapy was recently introduced as a novel therapeutic modality that significantly extends GBM patients’ life. The treatment requires the chronic placement of transducer arrays that generate alternating electric fields on the patient’s head. Recent studies suggest that increased electric fields intensity is associated with better treatment outcomes (Ballo et al., 2019; Glas et al., 2022). TTFields treatment planning requires the segmentation of GBM tissues on post-operative MR images for evaluating the distribution of TTFields in the tumor. Segmentation of post-operative GBM also facilitates quantitative follow-ups of disease progression and treatment efficacy evaluation. Manual segmentation of post-operative GBM on a typical T1w Gad MRI image is time consuming, i.e., 20–50 min for a highly skilled and experienced annotator.

A dataset of annotated pre-operative GBM MR images is publicly available through computational-challenges and other platforms (Menze et al., 2015; Bakas et al., 2018; Pei et al., 2020). As a result, many studies have investigated the segmentation of pre-operative GBM with results that are comparable to those of human raters (Wadhwa et al., 2019). However, annotated post-operative GBM MR images are much less studied. Moreover, it is unclear if the methods that were developed for pre-operative GBM work well on post-operative datasets. Few investigators have developed methods for post-operative GBM segmentation in the past decade (Cordova et al., 2014; Meier et al., 2014; Henker et al., 2017; Chang et al., 2019; Ermiş et al., 2020). Most of the studies focus on extracting clinically relevant measures that can tolerate some inaccuracies in the segmentation (Cordova et al., 2014; Henker et al., 2017; Chang et al., 2019). A method for an automated brain resection cavity delineation is presented in Ermiş et al. (2020). Meier et al. (2014) demonstrate a method for fully automatic semi-supervised learning for post-operative brain tumor segmentation by fusing information from both pre- and post-operative image data. The large amounts of data and data annotations available today, result in noisy labeling, and intra- and inter-observer variability. Thus, uncertainty has been recently studied on natural and medical images (Egger et al., 2013; Cordeiro and On, 2020; Karimi et al., 2020; Tajbakhsh et al., 2020). For example, Egger et al. (2013) studied the feasibility of incorporating 3D Slicer for reduced-time semi-automatic segmentation of GBM to follow-up the tumor volume and other treatment outcomes. They reported an average inter-observer Dice coefficient of ∼88% (range 76–96%) and average Hausdorff distance of 2.3 mm (range 0.31–3.7 mm). One important conclusion is that since various annotators may segment the same object differently, an intuitive tool for editing the segmentation results is required. Moreover, visualization of the segmentation uncertainty can be utilized to improve the trust of annotators in the quality of the methods (Nair et al., 2020; Mehta et al., 2022).

In this study, we present a novel method for post-operative GBM segmentation. Our method includes the surgery type, the cerebrospinal fluid (CSF) segmentation, and calculates segmentation uncertainty. We incorporated the uncertainty model in a custom intuitive segmentation editing tool and gathered feedback from three physicians. Then, we compared the accuracy of the proposed method to that of the current state-of-the-art. Last, we evaluated the usability of our method for TTFields treatment planning. The results indicate that the proposed method can significantly reduce the processing time of GBM segmentation while achieving a clinically acceptable segmentation model of the tumor. In addition, the uncertainty model may assist in studying the variance between experts’ annotations and developing a method that adjusts to the specific expert segmentation preferences. For example, if an expert consistently prefers over-segmentation that is less accurate, an adaptive method may adjust the segmentation results accordingly.

## Materials and methods

### Patients and data

**FIGURE 1:**
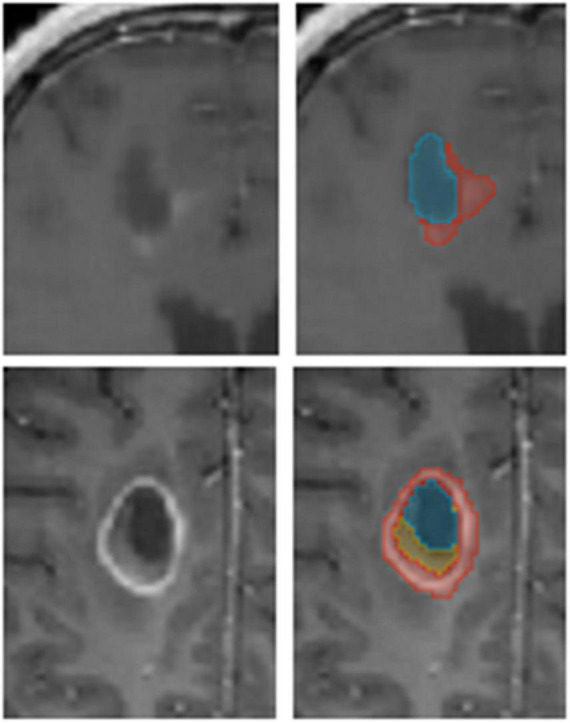
An example of annotated data. The glioblastoma multiforme (GBM) tumor as observed on MR T1 gad images **(Left)** along with annotators’ segmentation **(Right)**. Enhancing tissue (red), necrotic core (yellow), and resection cavity (blue) are segmented for Tumor Treating Fields (TTFields) treatment planning.

In this study, we evaluated the results of 340 GBM patients’ scans treated with TTFields. TTFields treatment planning was based on T1 gadolinium MRIs with a typical voxel size of 1 ×1 × 1-2 mm^3^. A trained team of annotators manually segmented the GBM tumor tissues on the MRI into three labels: (1) Enhancing tissue; (2) Necrotic core, and (3) Resection cavity (Figure 1). Note that we unify enhancing tumor and other enhancing appearance that may not be related to the tumor. For example, after a resection, the surface of the resection cavity appears as enhancing. Generally, we leave this decision to the expert physician. We were unable to delineate edema since it is much less prominent after surgery in the T1 imaging modality. A radiologist validated the labels and revisions were made as needed. An analysis of three experts’ annotations of 10 GBM MR images was performed in order to estimate inter-observer variability. Average Dice score of 0.72 (SD = 0.12; *N* = 30) was measured for the whole tumor segmentation between our annotators. Lower Dice values were observed on the specific tumor’s substructures. Resection was associated with 0.54 (SD = 0.07; *N* = 30) Dice coefficient, necrotic core with 0.57 (SD = 0.08; *N* = 30), and enhancing with 0.58 (SD = 0.08; *N* = 30). The observed Dice score is comparable, but somewhat lower than those reported in other studies (Lee et al., 2019). In other words, the Dice differences reported above represent the upper bound for Dice scores between the model’s prediction and ground truth. Better scores may be explained by a bias in the algorithm toward certain annotators.

### Neural network model design and implementation

We used nnUnet (Futrega et al., 2022), a model derived from Unet (Ronneberger et al., 2015) and tuned to segment brain tumors in MRI scans. This model demonstrates state-of-the-art results on pre-operative GBM segmentation tasks (Menze et al., 2015). Unlike the multi-modalities available in public datasets for pre-operative GBM segmentation such as the Brain Tumor Segmentation Challenge (BraTS) (Menze et al., 2015), only T1 enhanced MRI is usually available for post-operative GBM TTFields planning. The baseline-model performs poorly in this case (see the Section “Results”). Based on recent studies (Tajbakhsh et al., 2020), the model performance was improved by including meta-data such as tumor type. A post-processing step was developed to integrate surgery type into our segmentation pipeline. In general, there are three types of surgery: (1) total resection of the tumor; (2) partial resection; and (3) biopsy of the tumor. In cases where the type of surgery cannot be determined, partial resection is used. Moreover, since both: the resection cavity and the ventricles contain CSF and are associated with the same image intensities, distinguishing the boundary between them also poses a challenge for human annotators. Hence, we decided to incorporate segmentation of the ventricles into the model to resolve this ambiguity. By co-segmenting the ventricles, we expect the mislabeling of resection cavity voxels to be reduced.

**FIGURE 2:**
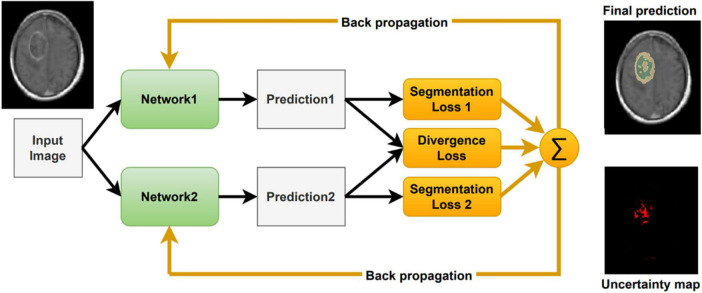
Our model architecture incorporating uncertainty as computed by the divergence loss.

Inspired by Wei et al. (2020), we include a segmentation agreement loss to cope with noisy labels and to facilitate an interactive visual review of the segmentation result (Figure 2). As in Wei et al. (2020), we jointly train two networks simultaneously. These networks share an identical architecture but differ in their parameters. Each network has its own segmentation loss with respect to the ground truth labels. Moreover, we calculate the divergence loss between the predictions of the two networks, so that the entire system can be jointly trained and updated. The proposed loss function for a given computed segmentation set *x*_*i*_ and ground truth one *y*_*i*_ is defined as follows:


(1) $$L_{t\,o\,t\,a\,l}=\left(1-\lambda \right)\;L_{s\,e\,g}\;\left(x_{i},y_{i}\right)+\lambda \,L_{d\,i\,v}\;\left(x_{i}\right)$$

where λ is a parameter that balances between the individual segmentation loss, *L*_*seg*_, and the divergence loss, *L*_*div*_. λ is set to 0.1. In our implementation, the segmentation loss is a combination of Dice coefficient and Cross Entropy as follows:


(2) $$L_{s\,e\,g}=\sum_{k=1}^{K}\left[\left(1-\alpha \right)\;L_{D\,i\,c\,e}\;\left(p_{k}\;\left(x_{i}\right)\;,\;y_{i}\right)+\alpha \,L_{C\,E}\;\left(p_{k}\;\left(x_{i}\right)\;,\;y_{i}\right)\right]$$

where *K* is the number of baseline models, currently set to 2. *p*_*k*_ is the prediction output of each baseline model. α is used to balance between Dice and cross entropy loss, currently set to 0.3. We empirically tested a few values of these hyper-parameters, training a model with a different set, and used the one that generated the best results. We did not conduct a thorough sensitivity test on these values.

As described in Wei et al. (2020), the two different networks tend to agree on prediction in the correct labels and disagree on incorrect ones. Therefore, this co-regularization term can guide the networks to a more stable model. The divergence loss measures the match between the two networks’ predictions and co-regularizes both networks. As proposed in Wei et al. (2020) we use a symmetric Kullback-Leibler (KL) Divergence loss as follows:


(3) $$L_{d\,i\,v}=D_{K\,L}\left(p_{1}||p_{2}\right)+D_{K\,L}\left(p_{2}||p_{1}\right)$$

In addition, to properly handle noisy labels, we follow the small-loss criterion proposed in Han et al. (2018); Ren et al. (2018), and Wei et al. (2020). This criterion is based on the idea that small loss samples are more likely to be correctly labeled. Therefore, in each mini-batch we sort the voxels according to their joint loss *L*_*total*_, and average over a part of the voxels with smallest values. This way, the noisy labeled voxels have a lesser effect on the total loss and the back-propagation update. In this study we presumed 90% as the percentage of correctly labeled samples in the dataset. The small-loss criterion is utilized for the divergence and cross-entropy losses that are calculated voxel-wise.

**FIGURE 3:**
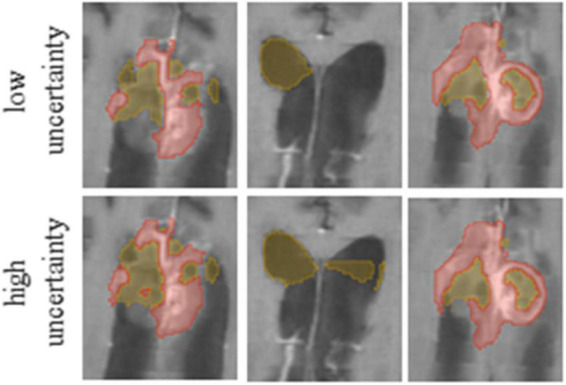
It is possible to edit segmentation results by throttling the uncertainty values. Compare the results for low **(Upper row)** and high **(Lower row)** uncertainty thresholds.

Our method averages the *softmax* operator on the predictions of the *K* baseline networks to compute the segmentation. According to Weijs et al. (2010) the KL divergence–considered as the relative entropy between two probability distributions, can be viewed as the uncertainty between these models. To visualize the uncertainty, we calculate the KL divergence between the baseline networks predictions. We normalize the uncertainty values over the dataset, such that the maximum uncertainty is set to 1 and the minimum is set to 0. We then present the uncertainty map as a heat map on top of the segmentation. Finally, we can utilize the uncertainty map to remove voxels that are associated with high uncertainty from the segmentation map, with a user defined threshold (Figure 3). Recent surveys (Gawlikowski et al., 2021) claim that there are multiple possible sources for uncertainty which, can be classified into: variability in real world situation, error and noise in the measurement system, and errors in modeling and training. By fixing the model and training method, we focus the uncertainty-estimate to the real-world situation variability and measurement system errors.

### Experimental setup and validation

We divided the set of 340 labeled T1 enhanced MRI brain scans of post-operative GBM patients to 270 training images and 70 images for test. All the images were resampled to 1 × 1 × 1 mm^3^ voxel size. As a pre-process we apply a bias field correction and a custom skull stripping method using the Advanced Normalization Tools (ANTs) (Tustison et al., 2010; Avants et al., 2014). During training we used a patch size of 128 × 128 × 128 voxels and apply the following augmentation on the data samples to increase the variability and improve model robustness: intensity normalization, random shift up to 0.1, random scale up to 10% of intensity values, and random flip of the image patch. We ensure a balance between patches that contain the tumor and those without it. We implement and train our model using PyTorch (Paszke et al., 2019) and MONAI^1^ with Adam optimizer (Kingma and Lei Ba, 2015) and 200 epochs. The learning rate was initially set to 1e-4 and gradually reduced whenever the metric comes to a plateau of 10 epochs.

In this study, we compared five models that were trained on the post-operative GBM training set: 1.a baseline nnUnet network (Futrega et al., 2022) trained on three labels: (a) resection; (b) necrotic core, and (c) enhancing; 2. the baseline nnUnet network including surgical information as a post-process; 3. using the model in 2, and segmenting CSF as an additional label; 4. our full model with two baseline nnUnet networks incorporating the surgical type, the CSF and uncertainty regularization as described above, and 5. our full model as in 4, but incorporating a user defined certainty threshold. To this end, we developed a graphical user interface in 3D Slicer (Fedorov et al., 2012) that allows the physician to choose the level of certainty in each case separately. Note that the certainty map is computed on the entire brain, but masked in the application to reflect the certainty in the tumor region. The threshold is determined by an interactive visual inspection in a trial-and-error process. We guided the experts to select a threshold to the GBM segmentation for the specific application of TTFields treatment planning. We observed that the experts tended to prefer over-segmentation results that cover the entire tumor over results that under-segment parts of it. Three experts rated the quality of the segmentation with respect to the clinical application of TTFields treatment planning on 12 patients MR T1 Gad images.

In addition, we evaluated the relevance of the suggested uncertainty measure with respect to accuracy. Note that the accuracy measures are per image, while the uncertainty values are per voxel. To compare the values, we suggest two uncertainty values that are computed per image: (1) the percentage of voxels that are assigned an uncertainty that is above zero from the voxels that reside in a tumor’s tissue, and (2) the median value of uncertainties of the voxels that reside in the tumor’s tissue.

## Results

**FIGURE 4:**
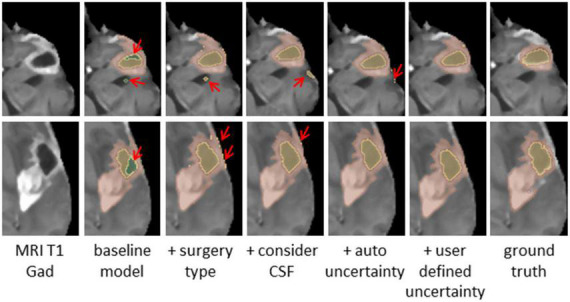
Glioblastoma multiforme (GBM) tumor as appeared on MR T1 gad images **(Left)** along with results of five models and the ground truth. The methods segment the resection cavity (green), necrotic core (yellow), and enhancing tissue (orange). Red arrows point to some of the visible errors in the segmentation results. These errors are reduced with our full method. The baseline model fails to distinguish between necrotic core and resection cavity, misconstruing some necrotic core areas as resection. By integrating surgery type, this issue is resolved. By segmenting the cerebrospinal fluid (CSF), some errors in labeling CSF as resection or necrotic core can be avoided. Our full model incorporating the uncertainty and simultaneous co-training of two models further reduces the visible errors.

Table 1 summarizes the Dice coefficient, Hausdorff and mean surface distances that were measured between the ground truth and the proposed method. We compared this model with the baseline model and other models that incorporate some or all features of our method (Figure 4). The Dice coefficients between our results and the ground truth are similar to the results observed between annotators. This indicates that our method achieved the inter-observer variability. Furthermore, although the baseline model achieved this state-of-the-art result on the whole tumor and the enhancing tissue, it was less accurate for the resection cavity and the necrotic core in comparison to our results. Table 2 illustrates the rate of mislabeling between different segmented structures. Our results show that the utilization of the surgery type and the segmentation of CSF dramatically reduce the confusion between CSF, resection cavity, and necrotic core. Fine tuning of the segmentation uncertainty further reduces the rate of mislabeling values.

**TABLE 1A:** A comparison of average Dice coefficient values for three glioblastoma multiforme (GBM) tissues and for the entire tumor segmented as one tissue.

	Resection	Necrotic core	Enhancing	Whole tumor
Baseline	0.59 (0.29)	0.55 (0.30)	0.66 (0.19)	0.8 (0.12)
Above + surgery type	0.64 (0.27)	0.6 (0.27)	0.66 (0.19)	0.8 (0.12)
Above + CSF	0.7 (0.23)	0.62 (0.27)	0.67 (0.2)	0.81 (0.11)
Above + auto uncertainty	0.7 (0.25)	0.63 (0.26)	0.67 (0.2)	0.81 (0.11)
Above + user def uncertainty	0.71 (0.24)	0.64 (0.25)	0.68 (0.19)	0.81 (0.11)

Each row extends the previous row model with another feature.

**TABLE 1B:** A comparison of mean surface distance (mm) for three glioblastoma multiforme (GBM) tissues and for the entire tumor segmented as one tissue.

	Resection	Necrotic core	Enhancing	Whole tumor
Baseline	6.8 (8.4)	5.12 (11.84)	3.46 (3.98)	2.64 (4.44)
Above + surgery type	6.45 (8.01)	6.61 (11.67)	3.46 (3.98)	2.63 (4.44)
Above + CSF	5.39 (8.18)	5.96 (12.26)	3.04 (4.2)	2.27 (4.88)
Above + auto uncertainty	4.95 (7.74)	5.42 (11.38)	2.97 (3.44)	2.19 (4.31)
Above + user def uncertainty	4.22 (6.13)	5.32 (11.04)	2.99 (3.57)	1.97 (2.62)

Each row extends the previous row model with another feature.

**TABLE 1C:** A comparison of Hausdorff distance (mm) for three glioblastoma multiforme (GBM) tissues and for the entire tumor segmented as one tissue.

	Resection	Necrotic core	Enhancing	Whole tumor
Baseline	51 (44.7)	19.49 (18.27)	38.71 (35.81)	33.63 (40.24)
Above + surgery type	51 (44.8)	34.42 (30.33)	38.71 (35.81)	33.63 (40.24)
Above + CSF	56.87 (45.89)	29.37 (31.14)	37.25 (33.68)	38.05 (40.99)
Above + auto uncertainty	39.9 (38.98)	28.65 (27.51)	36.4 (33.54)	32.25 (38.34)
Above + user def uncertainty	33.63 (35.17)	28.61 (27.03)	35.54 (32.9)	29.56 (35.94)

Each row extends the previous row model with another feature.

**TABLE 2:** Rate of mislabeling comparison.

	CSF as resection	Necrotic core as resection	Resection as necrotic core
Baseline	0.45	0.34	0.48
Above + surgery type	0.09	0.05	0.07
Above + CSF	0.04	0.06	0.07
Above + auto uncertainty	0.04	0.06	0.07
Above + user def uncertainty	0.03	0.05	0.06

The baseline model demonstrated a reoccurring error between cerebrospinal fluid (CSF), resection, and necrotic core. Using our method reduces this error.

**FIGURE 5:**
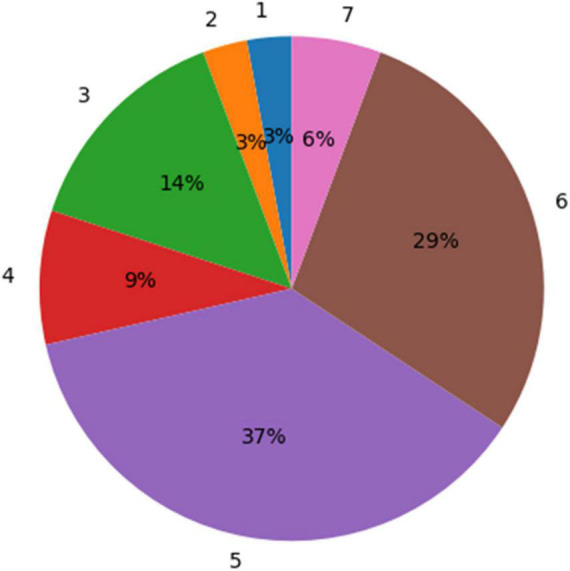
A distribution of the experts’ ratings for the glioblastoma multiforme (GBM) segmentation using the uncertainty values. Seventy-two percent of the segmentation results were rated “acceptable as is for treatment planning” (5) or better (scores 6 and 7).

According to the physicians’ ratings, 72% (26 out of 36) of the GBM segmentations were found adequate for TTFields treatment planning (grades 5–7; Figure 5). whereas, for the remaining cases, 22% (rated 3–4) required a short manual edit and the rest 6% of cases require a significant editing before further considering them for this application. More specifically, the most common comment in cases of score of 1–4 was under-segmentation of the resection cavity or the necrotic core. When the segmentation was adequate, it took the physician approximately 2 min to select the uncertainty threshold and validate the segmentation on the T1 enhanced MR image. This suggests a significant reduction of segmentation time compared to the typical 20–50 min manual GBM segmentation.

**FIGURE 6:**
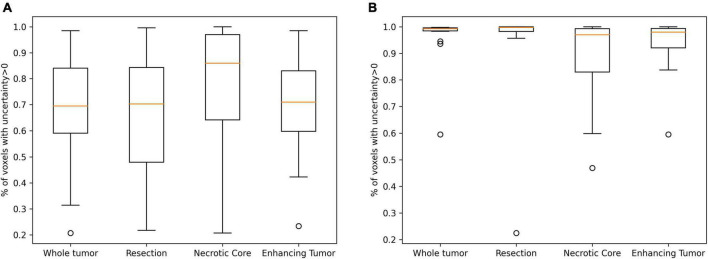
A comparison of uncertainty values of high (**A**; Dice > 0.5) and low (**B**; Dice < 0.5) accuracy segmentations. Lower accuracy segmentations were associated with higher uncertainty values.

Table 3 summarizes the Spearman correlation between the uncertainty and the accuracy. The two suggested uncertainty measures demonstrate a significant correlation (*p* < 0.05; after multiple comparison correction) with the “resection” and “enhancing” tumor tissues segmentation accuracy that was measured with the Dice coefficient. The median uncertainty measure demonstrated lower inverse-correlation with the Dice coefficient in comparison to the other uncertainty measure of percentage of voxels that are assigned above zero voxel-uncertainty. A comparison of uncertainty values of high (Dice > 0.5) and low (Dice < 0.5) accuracy segmentations suggests that lower accuracy segmentations were associated with higher uncertainty values (Figure 6).

**TABLE 3:** Spearman correlation between uncertainty and accuracy measures.

	Median voxel	% of voxels > 0
Whole tumor	−0.39*	−0.27*
Resection	−0.64*	−0.49*
Necrotic	–0.24	–0.10
Enhancing	0.62*	−0.40*

Image-uncertainty was estimated by two methods: (1) median voxel-uncertainty value, and (2) percentage of voxels above zero. Accuracy was measure with the Dice coefficient.

*p < 0.05 after multiple comparison correction.

## Discussion

The Dice coefficient, Hausdorff and mean surface distances demonstrate that the method have achieved state-of-the-art accuracy. Moreover, the Dice scores observed using our method are similar to these observed between raters. Therefore, in terms of Dice, this method has reached an expert level result. The observed rate of mislabeling values suggest that the utilization of surgical type and segmentation of CSF reduce the errors in segmentation of tissues within the GBM tumor. The physicians’ comments suggest two common errors that can be further handled. The resection/necrotic-core segmentation error can be further reduced *via* a graphical user interface that facilitates re-labeling of specific segments. Moreover, we will revise the model objective function to reflect the physicians’ low tolerance for under-segmentation, in comparison to over-segmentation. The physicians’ ratings suggest that 72% of the segmentations are adequate as is and another 22% can be briefly edited before integration in TTFields treatment planning. In these cases, the expected time reduction is 15–35 min per treatment planning. Therefore, the suggested method has a great potential to reduce the overall planning time or to increase the clinic throughput.

To conclude, we present a novel method for segmentation of GBM on post-operative T1 enhanced MRI scans. The method incorporates the surgery type and segments the CSF in addition to the GBM to improve its delineation. Moreover, we jointly trained two segmentation networks using a unified loss consisting of segmentation loss and divergence loss. This architecture can be extended to multiple networks and baseline architectures. In future work, more complex meta-data can be used in the training phase to improve the tumor labeling for different patients. Our final result is a segmentation map augmented with an uncertainty map, that can be further facilitated for fast and interactive selection of personal preference.

## Data availability statement

The original contributions presented in this study are included in the article/supplementary material, further inquiries can be directed to the corresponding author.

## Ethics statement

Ethical review and approval was not required for the study on human participants in accordance with the local legislation and institutional requirements. The patients/participants provided their written informed consent to participate in this study.

## Author contributions

MH designed and implemented the parallel network and the uncertainty score visualization, implemented its usability test in 3D Slicer, worked with the annotators to estimate the inter-observer variability, wrote the first version of the manuscript, and revised the last version. RF implemented a large-scale validation to compare the method for the specific task of TTFields treatment planning, adjusted the model’s hyper-parameters for better performance, produced some of the figures, and revised the manuscript. KS co-designed and co-executed the usability test, helped with its analysis, and revised the manuscript. OP co-designed the large-scale validation that is specific to TTFields treatment planning, supervised its implementation, and assisted with the analysis and manuscript revision. RS co-designed the parallel network, suggested segmenting the normal tissues along the pathology, led the design of the validation, and revised the manuscript. All authors contributed to the article and approved the submitted version.

## Footnotes

1
https://monai.io/
